# Dynamic relationship between multiple START assessments and violent incidents over time: a prospective cohort study

**DOI:** 10.1186/s12888-014-0323-7

**Published:** 2014-11-26

**Authors:** Richard Whittington, Johan Håkon Bjørngaard, Andrew Brown, Rajan Nathan, Stephen Noblett, Beverley Quinn

**Affiliations:** Institute of Psychology, Health and Society, University of Liverpool, Liverpool, UK; Department of Neuroscience, Faculty of Medicine, Norwegian University of Science & Technology NTNU, Trondheim, Norway; Forensic Department and Research Centre Bröset, St. Olav’s University Hospital, Trondheim, Norway; Department of Public Health and General Practice, Faculty of Medicine, Norwegian University of Science and Technology, Trondheim, Norway; Secure Division, Mersey Care NHS Trust, Liverpool, UK; CAMEO Early Intervention Service, Cambridgeshire & Peterborough NHS Trust, Peterborough, UK

**Keywords:** Violence, Risk assessment, Forensic psychiatry

## Abstract

**Background:**

Dynamic risk factors need to be assessed repeatedly over time rather than at a single time point to examine the relationship with violence. This predictive validity study sought to examine the degree of dynamic change in risk assessed in a group of mentally disordered offenders and the relationship between change and the occurrence of violence.

**Methods:**

Routine structured assessments of Strengths and Vulnerabilities on the Short-Term Assessment of Risk and Treatability (START) instrument (n = 475) were linked prospectively with 275 violent incidents using logistic regression in a sample of 50 patients.

**Results:**

Stability within patients estimated using the intra-class correlation coefficient was high (>.80) for both Strengths and Vulnerabilities. In the overall sample, a 10 point increase in START Vulnerabilities score was associated with a three-fold increased risk of violence (OR = 3.1; 95% CI, 1.47-7.46) but there was no association for Strengths score (OR = 0.91, 95% CI, 0.34-2.47). When examined within patients, both Vulnerabilities (OR = 1.77, 95% CI, 0.56-5.54) and Strengths (OR = 2.26, 95% CI, 0.38-13.42) were associated with an increased risk of violence but in both cases precision was low due to reduced sample sizes.

**Conclusions:**

Risk factors which are considered to have the capacity to fluctuate dynamically did not do so substantially in this group of mentally disordered offenders. When fluctuations did occur there was some tentative evidence that they are associated with violent outcomes and could guide the use of prevention measures.

## Background

The distinction between stable and dynamic risk factors in the structured approach to risk management is well-established and highly important in developing targeted interventions for risky individuals. Douglas and Skeem [1] differentiate the idea of a person having a relatively fixed risk *status* from that of being in a certain type of more-or-less temporary risk *state*. Risk status denotes an unchanging level of risk whilst the fluctuating nature of a risk state enables changes over time and situations to be considered in treatment planning. A focus on risk status prioritises risk assessment in terms of inter-individual ‘between persons’ variation to address the question ‘how risky is this person compared to others?’ Risk state adds to this an appreciation of intra-individual variation between and within-persons to ask ‘how risky is this person right now compared to others and compared to the past?’ If risk status is concerned with ‘who?’, risk state is concerned with ‘who when?’

The implication of adopting a focus on risk state is that risk assessments should be conducted repeatedly in order to track any potential changes over time. Since risk status is seen as a fixed aspect of an individual, one assessment will be sufficient to make predictions about risk and decisions about treatment over long time periods. In other words, dynamic risk assessments have short term validity whilst stable risk assessments are intended to have long-term applications and remain relatively unchanged between long term reviews. This time-limited function of dynamic risk assessment is acknowledged by both researchers and practitioners but most research on risk factors, whether stable or dynamic, is based on single point assessments and adopts a standard approach in examining the relationship between risk score and violent outcome i.e. inter-individual variation in terms of area under the curve (AUC) analysis [2]. Most mental health services do assess dynamic risk factors using repeated measurements as part of routine practice but the data from these repeated assessments seems rarely to be included in formal analyses of predictive validity [3].

A typical example is research relating to the Short-Term Assessment of Risk and Treatability (START) instrument [4]. This is an SPJ tool explicitly designed to focus on dynamic risk factors with 20 items rated in terms of their contribution as potential Strengths and Vulnerabilities to the individual and an overall specific risk estimate (Low, Medium or High) for a variety of target behaviours including risk to others, self-harm and unauthorised leave. These estimates are informed by, rather than calculated from, a composite of the individual item evaluations. Items can be highlighted for particular attention as ‘key’ factors on the Strengths scale and ‘critical’ factors on the Vulnerabilities scale and there is scope to note imminent danger under a separate ‘THREAT’ item. Assessments are based on case note reviews and discussion amongst multidisciplinary teams to establish the overall risk of various adverse events, including violence. The focus is on short-term risk assessment and evaluations can be conducted weekly if necessary. The instrument was developed from 2004 onwards primarily within forensic mental health settings but with intended applicability to other types of psychiatric service.

Given the short time since its inception, there is a relatively substantial empirical literature on the properties of the START [5-10]. This includes examination of its implementation, clinical utility [11,12], internal consistency and inter-rater reliability [13]. Evidence of its predictive validity in seven studies has been summarised by Chu et al. [14] with AUC estimates for Vulnerabilities ranging from 0.66 to 0.83 and those for Strengths ranging from 0.65 to 0.77. However, despite the inherently dynamic nature of the instrument, all of these studies adopted the standard approach of calculating the relationship between an initial START score at a single time point and violence at a small number of subsequent points in the future (e.g. one year, five years and ten years). As a result the dynamic nature of the START is ignored and there is no attempt to examine how changes in risk factors are associated with changes in violence risk. This oversight is a common feature of the general risk assessment field, not just a problem with START research [15].

Wilson et al. [16] noted this problem with existing research and adopted a somewhat more dynamic approach by estimating the relationship between START scores at multiple baselines and subsequent violence at multiple endpoints. They found that “changes in dynamic risk factors were significantly associated with institutional violence, even after controlling for static risk factors”. However, the five time periods in this study were still relatively lengthy at three months when it is clear that many dynamic factors fluctuate much more rapidly, over days and even hours. There was also no attempt to examine intra-individual variability in this study.

The study reported here was designed to go one step further and examine the dynamic relationship between START scores and violence over periods of 30 days. Furthermore, it was intended to examine the variability in degree of dynamic changes between individual patients. Since some patients may be stable overall and others may be highly variable in their presentation, intra-individual variation in these fluctuations and their relationship to violence were examined.

### Aims of the study

- to track changes in risk over time in a group of mentally disordered offenders;
- to estimate the variability in risk score that could be attributed to stable patient characteristics versus dynamic changes within patients during follow up; and
- to establish the relationship between change in risk factors and the risk of violence

## Methods

The study took place on a Medium Secure Unit in England following approval by the North West England 1 Research Ethics Committee. The unit provides care and treatment to males and females who present a danger to the public due to mental illness but who do not require high secure care. It has 66 beds on six single-sex wards (one female).

The START was introduced into routine practice in 2008 with an intention for completion on every patient every two weeks to inform multidisciplinary team discussions. Assessments were conducted by all qualified nurses working in the unit at the time and some patients were assessed repeatedly by the same nurse. The assessments contributed to treatment and discharge planning but the final decision about discharge was taken by the responsible medical clinician. All START assessments completed from October 2008 to January 2011 (27 months) were eligible for inclusion in the study. Over this period there were 110 admissions to the unit. Assuming the 66 bed unit had permanent full occupancy for the entire 27 month period and two assessments were conducted per month as per the local procedure, 3,564 assessments should have been conducted.

Ultimately, 540 records were provided to the research team which represent 15.2% of all potential assessments. Furthermore, only 475 of these assessments (13.3%) achieved a threshold of having 14 or more completed items and were included in the study. Where data on up to six individual items were missing, scores for the missing items were imputed from the mean score for all completed items and added to the total for completed items to produce an imputed total score. Assessments with seven or more missing items were excluded from the analysis. As approved by the Research Ethics Committee, patient consent for data analysis was not required as it was based on anonymised routine clinical data.

The 475 included assessments were conducted on 50 patients (45.4% of admitted patients). The median number of START assessments was 8.5 (IQR = 10.8) with a range from one to 25. Five patients (10.0% of the sample) accounted for 22.3% of assessments (n = 107 assessments). The median number of days between START assessments was 22 (IQR = 20.1) but in some cases there was up to four months between assessments. The median from admission to the first START assessment was 312 days (IQR = 563.0) but this varied markedly from two to 1580 days (> four years), indicating substantial differences in mental state acuity on entry to the study. Data on index offences in the sample were not available.

### Data analysis

Descriptive statistics at each START assessment point (1–25) were calculated and the degree of change in discharged and non-discharged groups was compared. All statistical tests were two-directional and adopted a 5% alpha level. Receiver Operator Characteristic (ROC) analysis was conducted based on the first START assessment and on all assessments using SPSS Version 20. Then a multilevel linear repeated measures regression model was conducted with adjustment for diagnosis (schizoaffective diagnosis, schizophrenia or other diagnosis), age as a continuous variable and sex [17]. All regression analyses were performed in STATA 13 for Windows (Stata Corp., College Station, TX). As an indicator of clustering at the patient level an intra-class correlation coefficient (ICC) was used [17] as an estimate of the degree of score stability within patients. If, for instance, there was no concordance of START scores within patients the ICC would be 0 whereas if each patient scored the same value at each measurement, then the ICC would equal one. In order to test whether the degree of clustering of START scores within patients was greater than would be expected by chance alone a likelihood ratio test was used with a limit of p < 0.05.

The association of START Vulnerabilities and Strengths scores with risk of a subsequent violent incident was also analysed with logistic regression. Following each START assessment the occurrence of a violent incident before the next START assessment or (if none) within the next 30 days was noted. Firstly, sex-, diagnosis- and age-adjusted logistic regression analyses were conducted, only taking into account the clustering nature of the data. Secondly, fixed-effect (conditional logit) models were estimated in order examine the association of change in START scores within patients and the occurrence of a subsequent violent incident. The fixed-effect logistic regression model used information from patients discordant on violent incidents during follow up. This enabled analysis of START scores within the same patient, thereby controlling for background characteristics (observed and unobserved) that could have confounded the association between these scores and the risk of a subsequent violent incident [17]. The association between ten point increases in Vulnerabilities and Strengths and the occurrence of violence was examined. Precision was evaluated with 95% confidence intervals (CI).

## Results

Of the 50 patients, 44 (88.0%) were male and 43 (86.0%) had a diagnosis of schizophrenia. Their mean age was 38.6 years (range 19–65) and the median length of admission regardless of the study start or endpoint was 829.5 days (approximately 28 months, IQR 799.5, range 120–2297 days). Eighteen (36.0%) had been discharged by the study endpoint. Compared with the total number of admissions during the study period, the sample did not differ substantially in age or sex distribution, but the study sample had a higher proportion of patient with a diagnosis of schizophrenia (rather than schizoaffective disorder) than the overall unit population during the period.

Violence data for these 50 patients over the period up until November 2011 were obtained from the formal adverse incident recording system run by the health care trust. Violent incidents were defined as episodes of physical or verbal aggression, including property damage. There were 275 incidents involving 26 (52.0%) of the patients. The median number of incidents per patient for all patients (violent and non-violent) was 2.5 (IQR = 7.75) with a range of 0–58. Five patients (10.0% of the sample) accounted for 65.4% of incidents (n = 180 incidents).

### Clinical utility of the START

Before examining the key results, it is important to note some trends in terms of non-completed items since this gives some insight into the clinical utility of the START instrument. Some START items were systematically missed during completion by practitioners. Data on Medication Adherence and Material Resources were missing for more than 20% of Vulnerabilities and Strengths assessments. Conversely data on Emotions and Relationships were missing for less than 10% of Strengths and Vulnerabilities assessments and data on Mental State, Social Support, Occupation and Insight were missing for less than 10% of Strengths assessments. The mean missing data rate per item was slightly higher for Vulnerabilities (16.0%) than for Strengths (12.4%).

### Overall changes in risk

**Figure 1:**
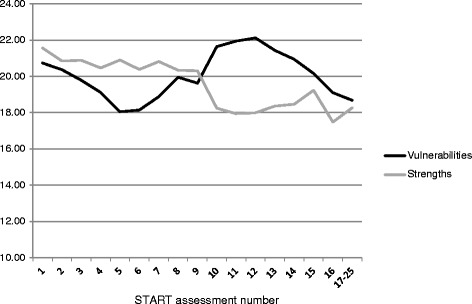
**Mean START score at each assessment.**

The mean scores (and standard deviation) in the first START assessment were 21.0 (8.6) for Vulnerabilities and 21.5 (7.5) for Strengths. Figure 1 illustrates the mean score for each of the two domains in a series from the first assessment point onwards for those points with assessments on 10 or more patients; data from assessment points with fewer than 10 patients (the 17^th^. onwards) have been aggregated. There was a 10% reduction in Vulnerabilities score across the combined sample as the number of STARTs (and thus the time in treatment) increased but this was accompanied by a 15% reduction in Strengths.

Those who had been discharged by the study end-date (January 2011) had a marked trend toward reduction in their Vulnerabilities score (mean = −3.9; 95% CI −7.9, +0.2) whilst those who had not been discharged at that point remained somewhat static (m = +0.1; −1.9, +2.1) but the difference in change between the discharged and in-patent groups was not significant. The discharged group also had a marked increase in their Strengths score (m = −4.0; +0.7, +7.3) whilst the in-patient group had somewhat reduced Strengths (m = −1.5; −4.5; +1.5). The difference in change between the discharged and in-patent groups on Strengths was statistically significant (=0.018).

### Predictive validity of the START instrument

A standard single-point ROC analysis of the predictive validity of the first START assessment and subsequent involvement in violence was conducted. Four patients were excluded from this analysis as their first incident occurred prior to their first START assessment. Twenty two (48%) of the remaining 46 patients were violent at least once subsequent to their first START assessment. The average period between the assessment and the first incident of violence was 231 days (sd 199 days, range 2–677 days). The AUC for Vulnerabilities was 0.69 (CI 0.52-0.85, p = 0.037) and that for Strengths (reversed for prediction of non-violence) was 0.75 (CI 0.59-0.89, p = 0.005). In addition a ROC analysis was conducted for all 475 START assessments across all patients to examine the relationship between START ratings and violence in the 30 days after the assessment. The AUC in this analysis was 0.74 (95% CI 0.64-0.84, p < 0.001) for Vulnerabilities and 0.55 (95% CI 0.47-0.64, p > 0.05) for Strengths (reversed for prediction of non-violence).

### Individual risk variability

**Figure 2:**
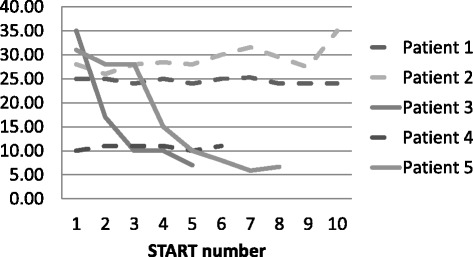
**Hypothetical individual variation in risk factor stability.**

The issue of differing patterns of stability is illustrated in Figure 2 with hypothetical data for 5 patients.

It can be seen that four of the hypothetical patients here are relatively high risk at their first assessment (Vulnerability score ≥25) and one is relatively low risk (score ~10). Patients 3 and 4 change rapidly and by their last assessment are scoring below 10 whilst Patients 1, 2 and 4 remain relatively stable in their risk level, even in some cases over 10 assessments. These varying individual patterns were then examined statistically in the study data using ICCs (see Table 1).

**Table 1:** **Intraclass correlation coefficients** (**ICC**) **of START Vulnerabilities and Strengths scores based on the results from multilevel linear regression models**

	**ICC**	**P**-**value** ^**b**^
START Vulnerabilities score^a^	0.84	<0.001
START Strengths score^a^	0.81	<0.001

^a^Adjusted for diagnosis group, age, sex and time in follow up.

^b^Based on a one sided likelihood ratio-test.

In the overall sample, about 84% and 81% respectively of the total variance of START Vulnerabilities and Strengths scores was attributable to the stability of scores within patients (ICC Vulnerabilities = 0.84 and ICC Strengths = 0.81).

Table 2 presents the associations between changes in START scores and the risk of a subsequent violent incident. In the total sample a ten point increase in START Vulnerabilities score was associated with increased risk (odds ratio, OR) of 3.1 (95% CI, 1.47-7.46). The within patients’ analysis from the fixed-effect logistic regression model uses information from patients discordant on violent incidents during follow up. A within patient ten point increase in START Vulnerabilities increased the odds of a violent incident with an OR of 1.77, but this estimate was made with low precision due to reduced power (95% CI, 0.56-5.54). The START Strengths score was not associated with the risk of a subsequent violent incident in the total sample (OR = 0.91, 95% CI, 0.34-2.47). In the within patient analysis, there was an increased risk of a violent incident (OR = 2.26, 95% CI, 0.38-13.42), but the confidence intervals were again wide in the fixed effect model due to the small sub-sample and none of these odds ratios were statistically significant.

**Table 2:** **Odds ratio with 95**% **confidence interval for a violent episode after START assessment** (<**31 days after assessment**) **in the whole sample and within patients** (**patient fixed**-**effect models**)

	**Total sample**			**Within patient association**		
	**OR**	**95% CI**	**P-value**	**OR**	**95% CI**	**P-value**
10 point START Vulnerabilities score increase	3.31^a^	1.47-7.46	0.004	1.77^c^	0.56-5.54	0.330
10 point START Strengths score increase	0.91^b^	0.34-2.47	0.857	2.26^d^	0.38-13.42	0.370

^a^Adjusted for diagnosis group, age, sex and time in follow up. 432 START risk assessments within 48 patients.

^b^Adjusted for diagnosis group, age, sex and time in follow up. 471 START strength assessments within 49 patients.

^c^Adjusted for time in follow up. 145 START risk assessments within 14 patients.

^d^Adjusted for time in follow up. 179 START strength assessments within 16 patients.

## Discussion

This study sought to examine the nature of dynamic risk in a sample of mentally disordered offenders by tracking changes in risk over time in the overall group and by examining the relationship between change in risk factors and the occurrence of violence. The essential question is: how stable or fluid are the risk factors which are generally considered to be dynamic?

In terms of context, the mean START scores at the first assessment indicate that the degree of risk presented by this group is comparable to that in other samples studied during the development of the instrument [11-14]. In addition, there is some evidence of tool validity here in that discharged patients had a substantially better change in risk factors (reduced Vulnerabilities and improved Strengths) compared to those who were not discharged in the study period. Some circularity is possible as discharge decisions will be based on a range of information sources of which START assessments are only one. Increased discussion of the potential for discharge based on these other sources (e.g. unstructured clinical judgement) could lead to assumptions by the assessor about improvement which then influenced START assessments in a form of self-fulfilling prophecy. However, the strength of the differences in START scores between the discharged and non-discharged group is preliminary support for the concurrent validity of the instrument. Also, in this setting, the nurses making the assessments would participate in discharge discussions as part of the multidisciplinary team but would not make the final discharge decision.

Predictive validity as estimated in the standard single point approach was acceptable in both domains and within the range of AUCs produced from other studies adopting this approach to analysis [14]. AUCs for Vulnerabilities in both ROC analyses were comparable to those obtained for other risk assessment tools examined in a recent systematic review [18]. The effect size for Strengths was also comparable for the first assessment predictions but not when all assessments were considered.

The key finding here is that risk factors which are considered to have the capacity to fluctuate dynamically did so only in a few patients, and did not do so in most of the patients included in this group. The ICC values for both domains were approaching 1, indicating that a very high proportion of variance in scores could be attributed to stability within patients. In addition there were mixed results in terms of whether change in risk score is associated with the occurrence of violence. A ten point (i.e. 25%) increase in START Vulnerabilities scores was associated with a threefold increase in the likelihood of violence over a four week period though this elevated likelihood was less than double when within patient associations were examined in the subsample with available data. Changes in Strengths score were not associated with any increased risk in the whole group although again risk was approximately doubled when the within patient association was examined. These within patient associations are the most comprehensive analyses but they are inevitably compromised by the small sample (~15 patients) with violent incidents in the relevant period which means that the estimates, however suggestive, are extremely imprecise.

The lack of fluctuation in risk may be accounted for in a variety of ways. The setting was a medium secure unit where the average length of stay is over two years. Therefore, whilst some participants will have been assessed shortly after admission from prison or the community, many of the patients will have been under treatment in the unit for a long period and thus their mental state will have been relatively stabilised. Alternatively, it may be that ‘staff cognition’ is not as dynamic as the tool itself. If staff have become somewhat de-sensitized to changing risk presentations or are drawn away from more regular patient contact they may miss the nuances of change in each individual. They may then assume stability and be blind to changes when conducting the assessment. This may be compounded by the 3-point scale for completion of each item which is relatively insensitive to small changes. Regular completion every two weeks furthermore will inevitably lead toward a ‘tick-box’ mentality where the primary task becomes completing the documentation rather than using the documentation as a tool to engage with the patient. The pattern of missing data also suggests some tendencies which may distort accurate completion. In particular, the assessments here were always completed by nursing staff and items which were regularly missed e.g. medication adherence, material resources, may have been considered as the responsibility of other members of the multidisciplinary team i.e. doctors and social workers.

## Conclusions

The main aim of this study has been to apply a new analytical approach to the study of dynamic violence risk. There are clear limitations in terms of the relatively small sample size, reliance on imputed data to fill gaps and the variability of timescales between START assessments and between admission and entry to the study. These limitations arise largely from the use of data collected in everyday practice for clinical rather than formal research purposes, a context which, on the other hand, increases the ecological validity of the assessment process. The clinical implications of the study are also limited as total scores were analysed rather than the summary risk ratings recommended by the instrument developers as the basis for clinical decisions. The next step is to address these weaknesses in data collection by conducting an adequately powered study with fully completed measures collected at regular time points. Such a study is under development and will enable a more valuable application of the approach to be conducted when it is completed.
